# Meeting the Aichi targets: Pushing for zero extinction conservation

**DOI:** 10.1007/s13280-016-0892-4

**Published:** 2017-01-31

**Authors:** Stephan M. Funk, Dalia Conde, John Lamoreux, Julia E. Fa

**Affiliations:** 1 Centro de Excelencia en Medicina Traslacional, Universidad de La Frontera, Piso 4, Av Alemania 0458, Temuco, Chile; 2 Department of Biology, Max Planck Odense Center, University of Southern Denmark, Campusvej 55, 5230 Odense M, Denmark; 3 Species 360, 7900 International DriveSuite 1040, Bloomington, MN 55425 USA; 4 11013 Ring Rd, Reston, VA 20190 USA; 5 Division of Biology and Conservation Ecology, School of Science & The Environment, Manchester Metropolitan University, All Saints Building, All Saints, Manchester, M15 6BH UK; 6 Nature Heritage, St. Lawrence, Jersey

**Keywords:** AZE, Endangered species, IUCN Red List, Protected areas

## Abstract

Effective protection of the ~19 000 IUCN-listed threatened species has never been more pressing. Ensuring the survival of the most vulnerable and irreplaceable taxa and places, such as those identified by the Alliance for Zero Extinction (AZE) species and their associated sites (AZEs&s), is an excellent opportunity to achieve the Aichi 2020 Targets T11 (protected areas) and T12 (preventing species extinctions). AZE taxa have small, single-site populations that are especially vulnerable to human-induced extinctions, particularly for the many amphibians. We show that AZEs&s can be protected feasibly and cost-effectively, but action is urgent. We argue that the Alliance, whose initial main aim was to identify AZEs&s, must be followed up by a second-generation initiative that directs and co-ordinates AZE conservation activities on the ground. The prominent role of zoos, conservation NGOs, and governmental institutions provides a combination of all-encompassing knowhow that can, if properly steered, maximize the long-term survival of AZEs&s.

## Introduction

Human impact on the environment has reached unprecedented levels. The planet’s biological–ecological, physical and chemical systems are threatened and with it our livelihoods (Stern and Treasury 2006; Rockström et al. 2009; Watson et al. 2016). On entering the Anthropocene (Crutzen 2002; Steffen et al. 2007) at least three of nine planetary boundaries have exceeded safe levels: climate change, global nitrogen cycle and integrity of biodiversity (Rockström et al. 2009; Newbold et al. 2016).

Based on a conservative estimate, a total of 477 vertebrates have vanished since 1900, over three-quarters of the 617 vertebrates that have become extinct since 1500 (Ceballos et al. 2015). Nonetheless, according to the most recent IUCN Red List of Threatened Species more than 7978 species of fishes, amphibians, reptiles, birds and mammals are globally threatened (IUCN 2016). Given these numbers, distinguishing which of these taxa to attend to first, and what resources to mobilize to ensure their survival, has never been so pressing (Wilson et al. 2011, 2016). Despite this urgency, progress has been slow. During the last two decades global strategies focussing on biodiversity and species conservation have concentrated on large-scale prioritization approaches (Redford et al. 2003; Brooks et al. 2015). But, these exercises have done little in terms of identifying the actual sites where conservation needs to occur (Brooks et al. 2006; Howes et al. 2009; Funk and Fa 2010). Likewise, high-level declarations of intent, epitomized by nation states signing international conventions, routinely confirm the need to ensure the long-term survival of the world’s biological diversity, while the biodiversity crisis continues.

Since the early 1990’s, a number of worldwide treaties have been signed. Major international agreements such as the Kyoto Protocol linked to the United Nations Framework Convention on Climate Change committed its Parties by setting internationally binding emission reduction targets. The detailed rules for the implementation of the Protocol were adopted in Marrakesh, Morocco, in 2001. However, international initiatives promoting the conservation of biodiversity at the highest level, in particular the COP-6’s (the sixth Conference of the Parties to the Convention on Biological Diversity) declaration have fallen short of their intended targets. COP-6 committed countries “to achieve by 2010 a significant reduction of the current rate of biodiversity loss” but because of a lack of significant improvements in the state of biodiversity (Butchart et al. 2010; Adenle et al. 2015), new targets were developed during COP-10 in Nagoya, Aichi Prefecture, Japan. These, referred to as the Aichi Biodiversity Targets, are a set of 20 objectives (subsequently abbreviated as T1, T2, …, T20), to be achieved by 2020 (SCBD 2010). There are three targets of direct relevance for the conservation of biodiversity: T11 (protected areas), T12 (species) and T13 (genetic diversity of plant and animal domesticates) (Table 1). For this review, we excluded T13 as it focuses on domesticated and cultivated plants and animals.

**Table 1:** Aichi Targets directly linked with the conservation of terrestrial species and ecosystems and prognosis for 2020

During the tenth meeting of the Conference of the Parties (COP 10) in Nagoya, Aichi Prefecture, Japan, the Convention on Biological Diversity (CBD) adopted a Strategic Plan for Biodiversity 2011–2020 with the mission to “take effective and urgent action to halt the loss of biodiversity in order to ensure that by 2020 ecosystems are resilient and continue to provide essential services, thereby securing the planets` variety of life, and contributing to human well-being, and poverty eradication” (SCBD 2010). CBD parties include 196 Parties of which 168 are signing parties (SCBD 2016). The Strategic Plan includes a set of 20 Aichi Biodiversity Targets, subsequently abbreviated as T1, T2, …, T20. The targets are clustered into strategic goals, whereby each target contains several sub-targets. For applied conservation of terrestrial species and ecosystems, T11 and T12 are the key targets. T11 and T12 are bundled in Strategic Goal C, which aims at “improving the status of biodiversity by safeguarding ecosystems, species and genetic diversity”. The following list only includes those sub-targets that apply to non-domestic terrestrial species and protected areas, PAs
T11: Increased global coverage of ecologically representative protected areas, PAs a: Conserving at least 17 per cent of terrestrial and inland water areas: **A** b: Conserving at least 17 per cent of coastal and marine areas: **B** c: areas of particular importance for biodiversity and ecosystem services conserved: **B** d: PAs are ecologically representative: **B** e: PAs are effectively and equitably managed: **B** f: PAs are well connected and integrated into the wider landscape/seascape: **B**
T12: Reducing risk of extinction a: Extinction of known threatened species has been prevented: **N** b: The conservation status of those species most in decline has been improved and sustained: **R**
The prognosticated progress (**A**: likely achieved; **B**: positive, but insufficient progress; **N**: no progress; **R**: regress) towards the implementation of the Strategic Plan for Biodiversity 2011–2020 follows the assessment by the CBD Secretariat (SCBD 2014)

A mid-term assessment of the Aichi Targets, indicates that current efforts will be insufficient not only to achieve most targets by 2020, but also that pressures on biodiversity will continue to rise over this period (SCBD 2014). According to this assessment, only one sub-target, T11a ‘*conserving at least 17 per cent of terrestrial and inland water areas*’ may be met (Table 1). All others are likely to fail. It is doubtful that T11a on its own will be enough to maintain biodiversity and ecosystem services in the long term. This is because T11 does not account for insufficient protected area (PA) management, lack of representativeness, degazetting or degradation (Mascia et al. 2014; Watson et al. 2014; Butchart et al. 2015). Likewise, improving the conservation status of threatened species and preventing their extinction, as designated in T12a, will likely not be achieved (Table 1). Given this dire prognosis, finding ways to maximize the conservation of a significant number of highly threatened species is urgently required.

By focussing on species that are most at risk of extinction, it will be possible to improve the prospects of achieving the aspirations set out in T11 and T12. More particularly, in this review we make a case that species and sites listed by the Alliance for Zero Extinction (AZE) already provide the global conservation community with a realistic opportunity to contribute to the Aichi targets to support countries to comply with these targets and to achieve the spirit of Aichi by directly reducing biodiversity loss. AZE, an alliance of conservation groups, aims to identify and promote the protection of taxa that are highly vulnerable to human-induced extinctions and are restricted to single locations (Table 2). Habitat conservation within AZE sites and/or the in situ and ex situ augmentation of population numbers of AZE species may require relatively straightforward interventions. However, this potential has only been partially realized (Butchart et al. 2012; Hsu et al. 2014; SCBD 2014; Butchart et al. 2015). AZE species and sites, henceforth AZEs&s, constitute a first line of defence against predictable and preventable imminent species losses (Ricketts et al. 2005). As stated by Butchart et al. (2012), the effective conservation of all AZE sites is “by definition essential to achieve the CBD target of preventing extinctions of known threatened species*”*. The protection of AZEs&s has been included as a critical piece of the Convention for Biological Diversity (CBD), and confirmed by the signing of mutual collaboration agreements in 2010 and 2011. AZE sites are considered a crucial component of the Key Biodiversity Areas framework (Brooks et al. 2016; Juffe-Bignoli et al. 2016). Additionally, AZE sites have also been included as part of the Critical Habitat Protection indicator in the Environmental Performance Index (EPI). The EPI provides a gauge at a national government scale of how close countries are to established environmental policy goals. This proximity-to-target methodology facilitates cross-country comparisons as well as an analysis of how the global community performs collectively on each particular policy issue (Emerson et al. 2010).

**Table 2:** Alliance for Zero Extinction, AZE, species and sites

Presented to the academic community in 2005 (Ricketts et al. 2005), the Alliance for Zero Extinction, AZE, is a worldwide consortium of currently 98 global biodiversity conservation organizations and an increasing number of regional partnerships, currently in Brazil, Columbia, Mexico, India and Peru (AZE 2013a). Membership is open for all NGOs with a focus on the conservation of biodiversity. AZE collaboration focuses on three principles (AZE 2011): ∙ Development of site map and site list ∙ Identification of conservation needs and implementing agencies ∙ Develop and raise funds for conservation programmes
There are no minimum requirements for the level of contribution and there is no obligation to make financial commitments. All members can also work independently without co-ordination of their priorities with AZE. No lead organization exists, but the AZE’s Secretary, currently the American Bird Conservancy, co-ordinates the activities, including the web presentation
AZE’s main focus is to identify ‘trigger’ species, which are threatened by immediate extinction, and their associated sites (American Bird Conservancy 2005; Ricketts et al. 2005). The criteria for choosing AZE species are straightforward: ∙ Species must be listed in the IUCN Red List of Threatened Species as either Critically Endangered or Endangered ∙ More than 95% of the species’ population must be restricted to a single and thus irreplaceable site ∙ The species’ site must have a definable boundary within ecological conditions different from adjacent sites
AZE sites are those, which contain at least one AZE species. Because species extinctions are likely in these sites, protection is essential.
AZE species have been identified so far for mammals, birds, amphibians, some reptiles, conifers and, in the recent update, reef-building corals. Originally, 794 species were identified in 595 sites, but the numbers have now changed to 920 species in 588 sites (AZE 2013b). AZE vertebrates currently include 502 amphibians, 165 birds, 157 mammals and 17 reptiles. Reptiles are underrepresented in this list because of the absence of an IUCN global species assessment for this group at the time of the launch of the new AZE website in 2014

In this review, we provide a summary of the available evidence on the nature of AZEs&s and evaluate the existing gap between the desired and realized efforts for the conservation of AZEs&s. We then provide practical guidance on how the protection of AZEs&s can contribute to stemming the loss of biodiversity, in support of T11 and T12.

## Current protection status of AZE sites and species

AZEs&s are interlinked. On their own, AZE species represent extremes of threat and irreplaceability; two widely used metrics to denote species that are highly vulnerable to extinction (Brooks et al. 2006). Because AZE sites cover relatively small land areas, they are particularly vulnerable to biodiversity loss drivers such as climate change, invasive species, pollution and human-induced land use changes. Available data indicate that 25% of all AZE species will be affected by urban expansion and encroachment in the next two decades (Seto et al. 2012). The highest impact is expected in Central and South America; a worrisome fact since the majority of AZE sites occur in the New World.

Global species diversity is currently underrepresented within the existing network of PAs (Rodrigues et al. 2004; Jenkins and Joppa 2009; Butchart et al. 2012). This is also true of AZE sites since only half are legally protected, with just over a third fully contained within a gazetted PA (Butchart et al. 2012). Even within protected AZE sites, measures to conserve threatened species in them may be absent or inadequate (Hsu et al. 2014). To date, only Brazil, Colombia and Mexico have included AZE sites into their national biodiversity protection strategies (Lamoreux et al. 2015).

Additionally, the increase of the proportion of PAs that cover AZE sites has declined over time with their coverage expected to be only about 24% by 2020, an increase of no more than 1% since 2010 (SCBD 2014). A sobering statistic is that there are three times as many AZE taxa at the risk of extinction as are species known to have been lost within the same taxonomic groups in the last 500 years (Ricketts et al. 2005).

## What does a conservation focus on AZE species mean?

Almost one-fifth of extant vertebrate species are classified as threatened, ranging from 13% of birds to 41% of amphibians; a figure that is increasing (Hoffmann et al. 2010; Ceballos et al. 2015). This bias towards amphibians is reflected in the AZE species list where 63% of the 841 AZE vertebrates are frogs, toads and salamanders (Table 2). This presents a significant challenge since amphibians are not just affected by habitat destruction; there are numerous cases where habitat is protected but amphibians are still disappearing. The causes of these declines are complex, but chytridiomycosis, a disease caused by fungal pathogens, together with habitat loss are the most significant threats. Chytrid disease is associated with the loss of hundreds of amphibian species at global scale, currently representing the “greatest species conservation challenge in the history of humanity” (Gascon et al. 2007). This means that the protection of amphibians from extinction requires not just the conservation of landscapes but direct actions, including ex situ conservation interventions.

**Fig. 1:**
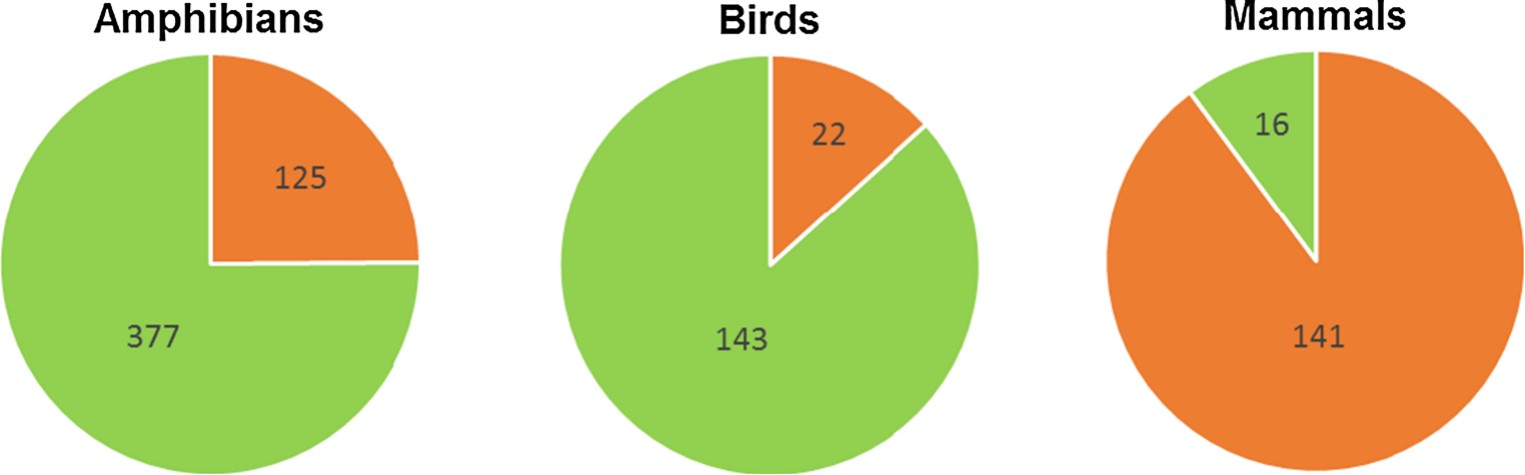
EDGE species amongst AZE mammal, bird and amphibian taxa. The EDGE score estimates evolutionary distinctiveness, thus irreplaceability, jointly with conservation status (Isaac et al. 2007). It increases with the degree of irreplaceability and conservation threat. EDGE species are the 100 highest-ranking amphibians, birds and mammals, respectively. Amongst AZE animals, mammals have the highest proportion of EDGE species (*orange*) and birds the highest proportion of non-EDGE species (*green*). EDGE data from Isaac et al. (2007) and the Zoological Society of London (2016)

The main justification for pursuing AZE species conservation is an ecocentric approach, in which nature’s intrinsic value is central (Butler and Acott 2007). However, it is possible to invoke additional arguments. In a comparison between ecosystem services in AZE sites and randomly selected sites, Larsen et al. (2012) found that the protection of AZE sites would result in the maintenance of ecosystem services, in turn generating direct human well-being benefits. Additionally, there are potential economic benefits from climate change mitigation at these sites. These benefits exceed the management cost of conserving AZE sites, delivering a disproportionate value for at least one ecosystem service in 89% of the sites (Larsen et al. 2012). Likewise, AZE species may contribute to the provision of potential future services e.g. new pharmaceuticals and other products (Gascon et al. 2015). Thus, if AZE species become extinct, this potential vanishes. Not only are the AZE species unique by definition, but a substantial number of AZE species are also listed as Evolutionarily Distinct and Globally Endangered (EDGE) species (Isaac et al. 2007). A total 35% of all AZE species (amphibians 25%, birds 13%, mammals 90%) are also EDGE species (Fig. 1). EDGE identifies species that have a disproportionate amount of unique evolutionary history. They have few close relatives, who are often the only surviving member of their genus, and sometimes the last surviving genus of their evolutionary family.

## Can we save AZE species from extinction?

**Fig. 2:**
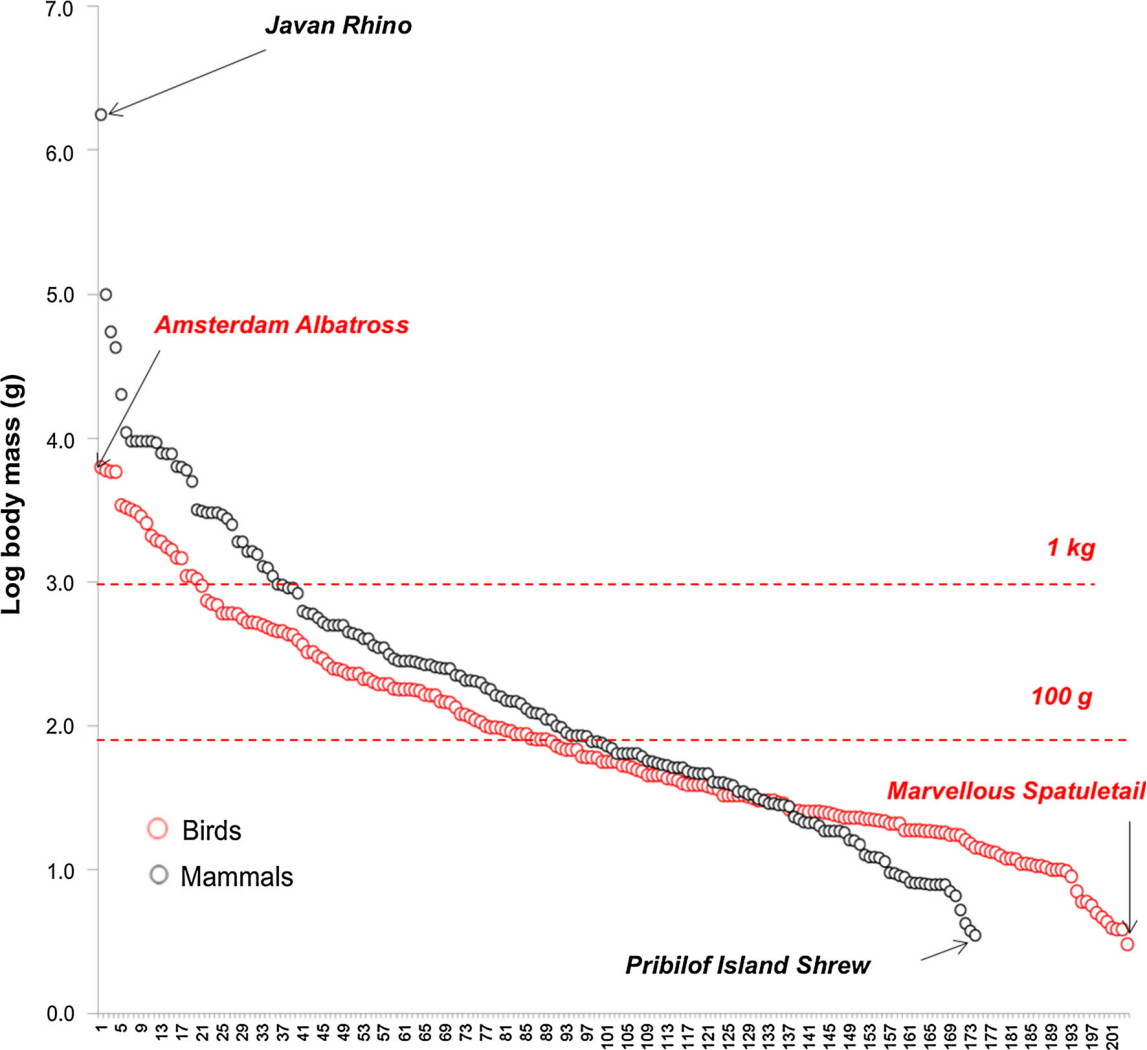
Body size distribution of AZE mammals and birds. Sizes are biased towards small and light birds, mammals with 92 and 79%, respectively, lighter than 1 kg, 65, and 56%, respectively, lighter than 100 g. All AZE amphibians and reptiles are lighter than 1 kg and are not shown here

It is clear that the AZE is an evolving project as species are added and some are lost to the list as they are declared extinct e.g. the Christmas Island pipistrelle (*Pipistrellus murrayi*) (Martin et al. 2012). Nonetheless, the remaining challenge is to protect and effectively manage AZEs&s, often in demanding geographical (rough terrain, remote sites) conditions, and restrictive geopolitical circumstances (corruption, insurgency, war) (Conde et al. 2015). However, AZE sites are relatively small (median size ~121 km^2^) compared to existing national parks and other protected areas (Ricketts et al. 2005; UNEP-WCMC 2016). This statistic, alongside the fact that most AZE species are relatively small-bodied animals (Fig. 2), may mean that despite the small area size of AZE sites, success is possible due to the universal relation of body size and landscape requirements (Thornton and Fletcher 2013). AZE species, being narrow-range endemics, generally inhabit reduced habitat spaces, and are thus less reliant on interconnected landscapes; management of wide-ranging species is much more difficult to achieve. Conservation of AZE species requires the protection of their sites, which we argue is relatively cheap if the political will at an international, national and local level exists. To this end, awareness building and a unified policy strategy by currently involved and to-be-involved organizations is crucial.

The conservation opportunity index (COI) was developed by Conde et al. (2015) to assess probability of success of in situ conservation of AZE species. The COI quantifies those factors that are likely to affect the likelihood of success: costs of land acquisition and management in the species’ range country, governance impediments to conservation including likelihood of political instability and politically motivated violence (including terrorism), and the impact of urban expansion on AZE sites. According to Conde et al. (2015), a total of 39% of AZE species have maximum COI with 80% being in the upper half and 3% in the lower quartile of the possible COI range. Therefore, a prioritization approach might be more effective by focusing on the species with higher conservation opportunities showed by the index.

## Costs and funding

**Fig. 3:**
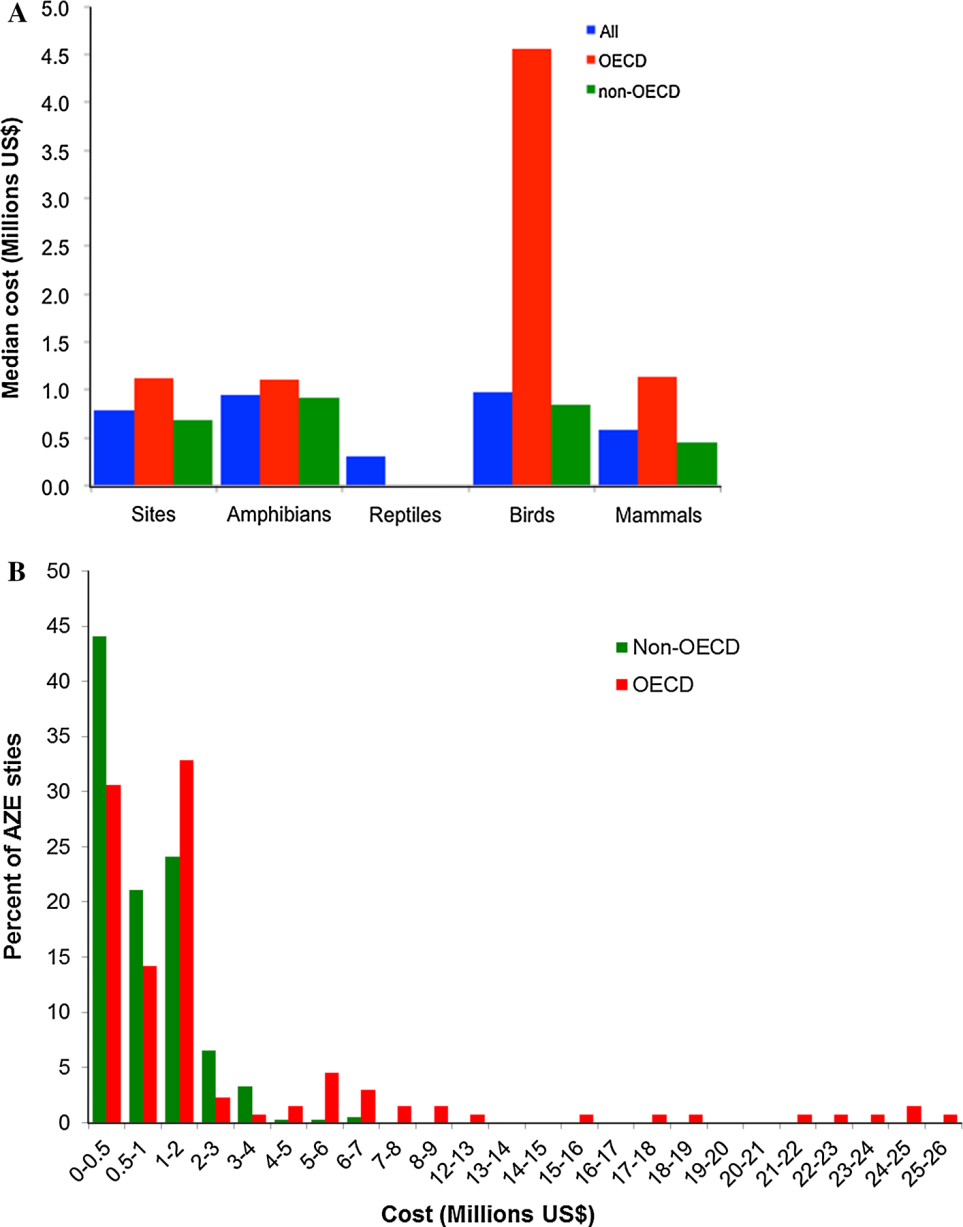
Total annual costs for conserving AZEs&s for sites where estimates are available (Conde et al. 2015). **A** Median cost for amphibians (*N* = 502), birds (*N* = 165), mammals (*N* = 157), reptiles (*N* = 17) and sites (*N* = 533) stratified whether sites are inside or outside OECD countries except for reptiles because of low *N*. **B** Site costs, ordered according to the values, for OECD and non-OECD sites

Annual costs for down-listing a threatened species on the IUCN Red List by at least one threat category have been estimated as ranging between $3.41 and $4.76 billion with or without considering shared expenditure between species (McCarthy et al. 2012). Based on the estimates of land purchase, area and habitat management, foregone monetary returns and transaction costs over a 20-year period, in situ protection for AZE species would require annual expenditures of between ~6000 and ~30 000 000 US$ (Wilson et al. 2011; Conde et al. 2015). Annual costs are highly skewed with the respective majorities at the lower end of cost and the minorities at the higher end (Fig. 3). Median costs for down-listing a species would be 0.94, 0.98, 0.58 and 0.3 million US$ for amphibians, birds, mammals and reptiles, respectively. These values are within the range of median annual cost values (0.04–8.96 million US$, average 0.85 million US$) estimated by McCarthy et al.’s (2012) to down-list threatened bird species by one threat category.

Costs for managing AZE sites are the same regardless of whether one or more AZE species are present (23% of AZE sites contain between 2 and 22 AZE species). It would cost around 791 million US$ per annum to manage all sites (Conde et al. 2015). As a proportion of a country’s GDP (estimated GDP for 2015 for OECD countries combined; (OECD 2016) the required annual expenditure for species and sites are low, from a minimum of 0.0016% to a maximum of 0.0024%. Management costs include both the establishment of protected areas and subsequent management of these sites, including those already under protection (Conde et al. 2015). Conservation costs vary according to the development status of a country. AZE sites in non-OECD countries, which contain ~60% of the AZE species, can be protected for less than in OECD countries (Fig. 3). As many as 65% of sites within non-OECD countries and 45% in OECD countries require less than one million US$ annually. The estimated median annual management expenditure per site in developing countries is 220 000 US$ (Conde et al. 2015).

## The need to incorporate AZE sites into the protected areas network

The current global PA network performs poorly in promoting the persistence of species, ecosystems, ecoregions or overall biodiversity (Rodrigues et al. 2004; Jenkins and Joppa 2009; Abellán and Sánchez-Fernández 2015). Crucially for AZE species, PA networks often do not adequately cover narrow-range endemics (Abellán and Sánchez-Fernández 2015).

Protecting AZE sites as government-protected areas is one strategy, but other forms of site protection should not be overlooked (Butchart et al. 2015). In many countries, lack of trust of public institutions is a strong barrier. Communities often do not have confidence in their own governments and, consequently, do not actively support legal protection; in some cases, they even boycott actions that come with the legal protection. AZE sites are, due to their generally small size, often easier to implement as community PAs compared to state PAs. Similarly, private ownership can significantly contribute to conservation, in particular as they can be efficiently managed, have high security, can quickly react to emerging threats and many have been shown to successfully achieve area protection, e.g. the Douglas Tompkins’ Pumalín Park in Chile. Community and private parks are, however, also exposed to risks such as a change of ownership, social-environmental conflicts and issues inherent to the tragedy of the commons (Holmes 2014, 2015). A recent analysis has shown that all types of area protection are powerful when applied in a regional mix of approaches (Leménager et al. 2014). Here, AZE sites offer opportunities to strengthen the mix and consequently achieve a stronger and more resilient PA system, provided that those potential risks arising from non-public ownership can be successfully addressed. The relative small size of AZE sites, flexible administrations and monitoring by third parties can turn formal protection into actual measures of conserving threatened species, a large problem for many formally protected large PAs (Hsu et al. 2014). AZE conservation must aim to have private and community sites embedded into the Alliance with adequate safeguards because the risks, such as change of ownership or economic use, can be severe and could obliterate the site and its species. Communal or privately managed or owned sites will not necessarily lead to the level of legal protection as required under T11a, but they can significantly contribute to several other sub-targets and overall strategic goal for which T11 stands. Whether or not communal- or private-protected sites contribute to T11, they directly contribute to T12 and these administrative models of AZE sites are a major opportunity.

## The role of zoos

The majority of institutions that compose the AZE consortium are zoos (AZE 2013a). Zoos work in the interface between ex situ and in situ conservation actions, now defined as the One Plan Approach, in which species are managed across different levels of human intervention from highly managed populations in zoos up to populations that are non-managed at all in the wild (Byers et al. 2013). Zoos are arguably the main institutions at a global level that have the knowhow and the experience for implementing scientific management programmes for the recovery of small animal populations and of rare species across this management continuum. This interface of population management is an integral part of the new Conservation Management Strategy of the World Association of Zoos and Aquaria (Barongi et al. 2015). Numerous examples where zoos have contributed successfully to the recovery of highly threatened animal taxa have been documented (see Fa et al. 2011). A number of zoos have singly or as part of conservation consortia made substantial advances in protecting AZE species. In a few cases, organizations such as the Durrell Wildlife Conservation Trust (Durrell), have worked on multiple AZE species, improving the protection of as many as 14 of them (two mammals, seven birds and five reptiles) as well as five other single-site taxa (Fa et al. 2011). For this organization, ex situ and in situ activities (albeit for a period of more than 50 years) have directly prevented the extinction of species such as the Mauritius kestrel (*Falco punctatus*). The conservation status of other taxa has been improved through the application of a suite of interventions such as fostering education, disseminating conservation science, raising countrywide attention to species in danger, and training local researchers, educators and managers (Aichi T1) as well as mobilizing funding resources (Aichi T1 and T20, respectively, Moss et al. 2015). Young et al. (2014) suggest that out of 17 target amphibian, bird and mammal species, eight underwent improvements in Red List category (reductions in extinction risk) owing to the conservation activities led by Durrell; a 67% increase in the value of the Red List Index between 1988 and 2012. This contrasts with a 23% decline in a counterfactual RLI showing projected trends if conservation had been withdrawn in 1988.

Despite a number of examples of success, zoological institutions need to step up their game if they are to remain relevant (Fa et al. 2014). Overall, most successful projects have resulted in the creation or management support of AZE sites by playing a crucial part in enhancing human livelihoods, which is an integral component of CBD’s vision to deliver benefits for all people by 2050 (Table 1). Thus, if one zoo-based organization has been able to improve the fate of a substantial number of AZE species, more involvement by zoos alongside other organizations could no doubt create a greater wave of direct and immediate conservation of AZEs&s. Although the role of zoos, and often their very existence, is still debated, they can play a vital role as emergency centres for species on the brink of extinction including amphibians. For the latter groups, zoos can actively engage in the ex situ breeding of rescued animals (in some cases the last of the species) for subsequent re-introductions if and when the conditions in the wild are adequate. Zoos and affiliated research centres can also generate the knowledge necessary to allow the treatment of the disease, raise awareness and engage in capacity building (Tapley et al. 2015).

In those cases, where AZE species require direct management by captive breeding as a safety net or a life-support system, costs for this would be relatively low; about 159 million US$ for all listed AZE vertebrates. AZE species are generally small-bodied animals (Fig. 2), and because of the positive correlation between ex situ cost to body weight (Fa et al. 2011) delivering effective captive breeding programmes, where needed, would be inexpensive. The estimated cost for amphibians and reptiles, are constant at ~10 000 US$ (Conde et al. 2015). Cost for birds vary moderately between 0.33 and 0.42 million US$ (median: 0.34) but much more for mammals, 0.66–10.27 million US$ (median: 0.38), reflecting the large size differences ranging from small rodents and insectivores to the Javan rhinoceros (*Rhinoceros sondaicus*). Although ex situ costs are relatively low, the decision to take this option must be based on a rigorous cost–benefit assessment and adaptive planning to maximize the chances of success (Fa et al. 2011; Tapley et al. 2015). In particular, the cost for re-introductions can be exceedingly high because this process and subsequent active management is lengthy and because achieving the optimal demographic and genetic structure of the new populations require well-planned long-term monitoring schemes.

## Recommendations for zoos

Besides the general difficulties of conserving biodiversity in the face of a sixth mass extinction (Ceballos et al. 2015), including the chasm between the legal and actual safeguarding of PAs (Watson et al. 2015), there are still several specific issues that affect AZEs. These include the need for a clear organizational setup, more targeted priority setting, planning and active involvement in conservation programmes on the ground as well as the plugging of existing financial gaps, and effective outreach and lobbying initiatives (Table 3). However, worldwide funding levels for conservation in general, and for PAs in particular, remains inadequate and undermine any efforts to meet the biodiversity targets (McCarthy et al. 2012; Waldron et al. 2013; Butchart et al. 2015). Major funding gaps for conservation are not limited to low-income countries but also extend to OECD nations, which have the highest incomes worldwide in terms of GDP (OECD 2016). For example, Chile is the ninth most underfunded country for biodiversity conservation worldwide and the lowest performing OECD member (Waldron et al. 2013), despite its outstanding biodiversity (Funk and Fa 2010). In general, however directing funds from high-income to low-income countries is a strategy that can bridge this financing gap (Waldron et al. 2013). For AZEs, zoos are especially well suited to achieve this goal due to their fundraising capacity and relatively straightforward administration. Thus, they are well placed to lead the Alliance’s fundraising activities towards AZEs&s. A major challenge is the expected increase in funding requirements of at least an order of magnitude to fulfil the world´s commitment to safeguard species and ecosystems by 2020 and beyond. New approaches such as crowd funding are starting to generate promising results and might bring new opportunities. Indeed, zoos support in situ conservation by fundraising and they already contribute 350 million US$ on conservation projects per year (Gusset and Dick 2011). Yet, much of the funds collected by zoos are directed not to AZE species but to high-profile threatened species and habitats and primarily mammals, in particular charismatic non-AZE primates and carnivores. Consequently, amphibians are significantly underrepresented (Gusset and Dick 2010). Finances in zoos are scarce, but much of these resources go to expensive species, which are less likely to face near-term extinction (Fa et al. 2011). Therefore, zoos must assess their commitment to AZE conservation and make an even stronger contribution to support their own AZE-related aims.

**Table 3:** Specific recommendations to transform AZE into AZE 2.0

Organizational infrastructure: AZE 2.0 ∙ Create a second-generation Alliance, AZE 2.0, suitable for efficient directing and co-ordinating active and efficient conservation of AZEs&s ∙ Establish the essential organizational infrastructure and funding of thereof ∙ Attract additional members strengthening and complementing the mix of expertise ∙ Create a web-based, open access platform for effective information dissemination to the public and as co-ordination tool between members.
Priority setting, planning and active conservation ∙ Joint development of a strategic, global and all-encompassing framework for the protection of AZEs&s ∙ Utilization of the wide geographic and disciplinary spread and expertise within all current AZE members and possible members of AZE 2.0 ∙ Refocus in the collection planning of the zoos committed to AZEs&s conservation ∙ Joint priority setting for in situ and ex situ conservation ∙ Critical analysis which species need and are suitable for ex situ breeding and which can be repatriated with a reasonable likelihood of success ∙ Long-term strategic planning ∙ Explore and support and monitor systems of protection and management that is most suitable for specific sites: private, community and state protection ∙ Identify research gaps for applied conservation, and co-ordinate, commission and implement research swiftly ∙ Proceed from academic and strategic planning to implementation swiftly
Fill financial gaps ∙ Utilizing the existing fundraising capacity of the AZE consortium, especially zoos, to support AZE-based conservation activities ∙ Encourage international multilateral organizations (e.g. GEF, World Bank, EU etc.) to put resources to promoting the conservation of AZEss
Outreach and lobbying ∙ Using the political leverage of the consortium to address the importance of AZEs&s ∙ Facilitate that AZEs&s are incorporated into national and regional conservation planning ∙ Utilize the available expertise amongst consortium members to extend focus in education, outreach and capacity building for AZEs&s

The priority setting approach by zoos, as best placed for mobilizing funds and conservation action for AZEs&s, remains a concern. Despite the fact that there is no doubt that zoos can play a significant role in in situ and joint ex situ conservation, support for AZE conservation is still rare (Fa et al. 2011). In our experience, zoo conservation actions are rarely used in a strategic, global and all-encompassing framework for biodiversity conservation and sustainable development. A recent horizon scan for zoos and aquaria identified the 10 most important emerging issues for species conservation by 2020, but AZE does not feature (Gusset et al. 2014). Clearly, a refocus is urgently required. First, participating institutions should focus more on keeping AZE species in their collections, based on critical assessments including priority setting, ex situ suitability, effects on demography and genetics, in situ conditions, and financials and logistic resources to maximize the chances of success (Fa et al. 2011; Pritchard et al. 2012; Conde et al. 2013). Second, the current gap in strategic priority setting for AZE needs to be addressed. There are numerous approaches on how to identify priorities for in situ conservation (Myers et al. 2000; Isaac et al. 2007; Funk and Fa 2010), ex situ strategies (e.g. Regional Collection Plans) and both combined (Fa et al. 2011; Byers et al. 2013; Conde et al. 2013), but a coherent prioritization scheme jointly amongst AZE partners still needs developing. It is difficult to encourage zoos to agree on a joint approach for prioritization even within regional zoo association species management programmes, let alone at an international level (Fa et al. 2011; Pritchard et al. 2012). Within institutions, prioritization in most cases relies on the preferences of directors, staff and visitors, but systematic planning such as the EDGE programme (Zoological Society of London, ZSL 2016; Isaac et al. 2007) is rare. Between in situ and ex situ organizations there has been a deep chasm in strategies and co-ordination (Fa et al. 2011; Pritchard et al. 2012). In situ and ex situ conservation programmes are often designed in isolation, not just of each other, but also of similar ones undertaken by others (e.g. other regions); a piecemeal approach that ignores the potential for making sure that ‘snowballing’ effects can be achieved (Fa et al. 2011). Only a unified system that can operate over large scales by drawing on spatially dispersed participants, e.g. across multiple conservation project sites, can result in an inter-communicated system of project sites that together can achieve cumulative change for a multitude of species and landscapes. Third, zoos can expand on their shift over the last decades from ex situ collections to in situ conservation and the new added pillar of education and outreach to underpin the societal value of zoos (Fa et al. 2011). This is a good foundation to focus efforts not only to concentrate on AZE species in their education programmes, but also to raise awareness for the urgent need of sustainable development and mobilize societal organizations for more active participation.

## AZE 2.0

In addition to the recommendations for the zoo membership of AZE, we have several recommendations for the Alliance itself. We put these under the banner of AZE 2.0. The current goals of the AZE focus on identifying AZEs&s, as well as their conservation needs and developing and funding programmes to protect them (AZE 2011). The first goal of identifying AZEs&s has been successfully accomplished (Ricketts et al. 2005) and fully revised (AZE 2010). However, it has become increasingly clear that the list of AZEs&s requires more frequent updates. Furthermore, the organizational structure of AZE should be modified if it is to achieve the identification of conservation needs portion of the vision. Perhaps most importantly, for AZE to reach its full potential as a widely recognized requirement for accomplishing Aichi T12, members will have to communicate and collaborate more closely, leveraging both money and expertise.

The AZE species list draws from the IUCN Red List of Threatened Species, which is constantly undergoing revision. Red List species accounts are updated to accommodate changes in our knowledge of species (e.g. distribution, taxonomy, population size, threats faced), to correct past errors, and to reflect genuine changes in conservation status. Correspondingly, the AZE list remains incomplete for terrestrial vertebrates because no global assessment for reptiles has been completed. Currently, the AZE list only includes 17 reptiles, but this list will likely increase given there are more than 10 000 lizards, snakes, turtles, crocodiles and tuatara worldwide (Böhm et al. 2013).

The necessity to update frequently the list of AZEs&s follows the need for updates to the Red List, but it also can require accounting for habitat/site changes on the ground. To date, the most thorough investigation of AZEs&s for a region found a considerable need for refinement (Lamoreux et al. 2015). AZE site delineation is particularly problematic. For example, Larsen et al. (2012) point out that only limited data exist for all AZE sites regarding boundaries. The Red List itself is also struggling to keep up with maintenance and revisions, which then weighs on the accuracy of the AZE list. Rondinini et al. (2014) stressed the importance of Red List maintenance and proposed steps by which to achieve this end. We follow this example by laying out the steps by which AZE can become more effective by tapping the resources of its membership organizations.

If we are able to harness and optimize the resources and expertise available across all members of the Alliance, we can advance effective conservation of AZEs&s. The Alliance has established an organizational infrastructure, which led to the successful identification of AZEs&s. Hitherto, this has been done as a volunteer operation. AZE members do not provide managerial or financial support to the Alliance, which means future updates to the data will remain infrequent. The Alliance is exploring the possibility of having countries identify and maintain their own AZEs&s list, which would feed into a global dataset (M. Parr, pers. comm.). However, we think it is time the member organizations step up by contributing dues to AZE. This would ensure regular data updates and allow the Alliance to pursue the conservation needs part of its original vision.

Compiling the conservation needs of each site is a different task from site identification. It requires ascertaining trends for a site, including its ownership, use and projected vulnerability to human disturbance, invasive species and climate change. It can also include a community profile and the identification of local partners who, if supported, would have the capacity and desire to conserve the site. Only such information will allow interested partners to judge which projects to launch, support or fund. Such an exercise is possible. BirdLife International has completed numerous detailed site profiles for their Important Bird and Biodiversity Areas Programme (Brooks et al. 2016). An in-depth study on the assessment of AZEs&s conservation needs was recently completed for southern Mexico (Lamoreux et al. 2015). The membership of AZE should support these types of data gathering, both financially and with their vast, collective expertise.

AZE 2.0 should also systematically capture the conservation outcomes (successes & failures), as well as the lessons learned from their efforts. Sharing data in this manner will improve the effectiveness of conservation actions. It will also allow the Alliance to measure and report on their impact; the information that is increasingly demanded by funders.

## Outlook

The target of reducing extinction risk, T12, is, by definition, immediately supported by the conservation of AZE species. For the target on PAs, T11, AZE sites’ contributions are differentiated according the sub-targets. AZE will contribute little to T11a’s 17% area size target and it is difficult to assess, due to data deficiency and lack of detailed analysis, how important AZE sites are for ecological representativeness, T11d, and the connection and integration into the wider landscape, T11f. On the other hand, they will contribute significantly to the other sub-targets. The contribution to sub-target T11c is immediate as it addresses sites of particular importance to biodiversity, to which the AZE species belong to as many of them also represent EDGE species, and it safeguards ecosystem services provided within and outside the sites. Due to their small size and the suitability for management as public, communal or private PAs, managed AZE sites will help to proceed towards achieving the sub-target on the effective and equitable management, T11e. The greatest impact of site protection is as a vehicle for T11. Additionally, the AZE approach also indirectly supports several other targets such as T1 (Awareness of biodiversity increased) and T20 (Mobilizing resources).

Conserving AZE-listed, highly vulnerable and irreplaceable species together with their associated sites will provide significant progress in the achievement of several Aichi targets and their underpinning goals, if the opportunities are acted upon. It is not only possible to conserve AZE sites and to prevent the extinction of many AZE species but inexpensive with a high likelihood of success (Conde et al. 2015). So far, the conservation potential has only been partially realized (Butchart et al. 2012; Hsu et al. 2014; SCBD 2014; Butchart et al. 2015).

Conservation is a long-term enterprise, requiring long-term monitoring and financing. Because AZE sites tend to be relatively small, they will likely be heavily affected by future climate change, hence making planning for climate change adaptation is necessary right from the start. If the AZE approach is to fulfil its potential for achieving targets 11 and 12, timely actions must be taken. From an economic point of view alone, swift action is required wherever possible to minimize the need for ex situ as the main conservation approach rather than providing “only” a safety net and to avoid the extra costs involved. A joint approach to protect and conserve AZEs&s needs to be finalized as soon as possible and a suitable organizational setup needs to be established allowing an efficient direction and co-ordination of the joint approach. This AZE 2.0 might arise from the current AZE or might be an entirely new platform, but speed is key.

## References

[CR1] Abellán P, Sánchez-Fernández D (2015). A gap analysis comparing the effectiveness of Natura 2000 and national protected area networks in representing European amphibians and reptiles. Biodiversity and Conservation.

[CR2] Adenle AA, Stevens C, Bridgewater P (2015). Global conservation and management of biodiversity in developing countries: An opportunity for a new approach. Environmental Science & Policy.

[CR3] American Bird Conservancy. 2005. Alliance for Zero extinctions. Pinpointing and Preventing Imminent Extinctions. Report. https://abcbirds.org/wp-content/uploads/2015/05/AZE_report.pdf.

[CR5] AZE. 2010. Sites & Species. *Alliance for Zero Extinction*. http://www.zeroextinction.org/maps/AZE_map_12022010.pdf.

[CR4] AZE. 2011. Memorandum of Understanding among all parties of the “Alliance for Zero Extinction.” http://www.zeroextinction.org/pdf/TermsofUseAZEdata_2011.pdf.

[CR6] AZE. 2013a. Alliance for Zero Extinction. *Alliance for Zero Extinction*. http://www.zeroextinction.org/index.html.

[CR7] AZE. 2013b. List of sites and species. *Alliance for Zero Extinction*. http://www.zeroextinction.org/sitesspecies.htm.

[CR8] Barongi R, Fisken FA, Parker M, Gusset M (2015). Committing to conservation: the world zoo and aquarium conservation strategy.

[CR9] Böhm M, Collen B, Baillie JEM, Bowles P, Chanson J, Cox N, Hammerson G, Hoffmann M (2013). The conservation status of the world’s reptiles. Biological Conservation.

[CR10] Brooks TM, Mittermeier RA, da Fonseca GAB, Gerlach J, Hoffmann M, Lamoreux JF, Mittermeier CG, Pilgrim JD (2006). Global biodiversity conservation priorities. Science.

[CR11] Brooks TM, Butchart SHM, Cox NA, Heath M, Hilton-Taylor C, Hoffmann M, Kingston N, Rodríguez JP (2015). Harnessing biodiversity and conservation knowledge products to track the Aichi Targets and Sustainable Development Goals. Biodiversity.

[CR12] Brooks TM, Akçakaya HR, Burgess ND, Butchart SHM, Hilton-Taylor C, Hoffmann M, Juffe-Bignoli D, Kingston N (2016). Analysing biodiversity and conservation knowledge products to support regional environmental assessments. Scientific Data.

[CR13] Butchart SHM, Walpole M, Collen B, van Strien A, Scharlemann JPW, Almond REA, Baillie JEM, Bomhard B (2010). Global biodiversity: Indicators of recent declines. Science.

[CR14] Butchart SHM, Scharlemann JPW, Evans MI, Quader S, Aric S, Arinaitwe J, Balman M, Bennun LA (2012). Protecting important sites for biodiversity contributes to meeting global conservation targets. PLoS ONE.

[CR15] Butchart SHM, Clarke M, Smith RJ, Sykes RE, Scharlemann JPW, Harfoot M, Buchanan GM, Angulo A (2015). Shortfalls and Solutions for meeting national and global conservation area targets: Meeting conservation area targets. Conservation Letters.

[CR16] Butler WF, Acott TG (2007). An inquiry concerning the acceptance of intrinsic value theories of Nature. Environmental Values.

[CR17] Byers O, Lees C, Wilcken J, Schwitzer C (2013). The One Plan Approach: The philosophy and implementation of CBSG’s approach to integrated species conservation planning. WAZA Magazine.

[CR18] Ceballos G, Ehrlich PR, Barnosky AD, Garcia A, Pringle RM, Palmer TM (2015). Accelerated modern human-induced species losses: Entering the sixth mass extinction. Science Advances.

[CR19] Conde DA, Colchero F, Gusset M, Pearce-Kelly P, Byers O, Flesness N, Browne RK, Jones OR (2013). Zoos through the Lens of the IUCN Red List: A global metapopulation approach to support conservation breeding programs. PLoS ONE.

[CR20] Conde DA, Colchero F, Güneralp B, Gusset M, Skolnik B, Parr M, Byers O, Johnson K (2015). Opportunities and costs for preventing vertebrate extinctions. Current Biology.

[CR21] Crutzen PJ (2002). Geology of mankind. Nature.

[CR22] Emerson J, Esty DC, Levy MA, Kim CH, Mara V, de Sherbinin A, Srebotnjak T (2010). 2010 Environmental Performance Index (EPI).

[CR23] Fa JE, Funk SM, O’Connell D (2011). Zoo conservation biology.

[CR24] Fa JE, Gusset M, Flesness N, Conde DA (2014). Zoos have yet to unveil their full conservation potential. Animal Conservation.

[CR25] Funk SM, Fa JE (2010). Ecoregion prioritization suggests an armoury not a silver bullet for conservation planning. PLoS ONE.

[CR26] Gascon C, Collins JP, Moore RD, Church DR, McKay JE, Mendelson JRI (2007). Amphibian Conservation Action Plan.

[CR27] Gascon C, Brooks TM, Contreras-MacBeath T, Heard N, Konstant W, Lamoreux J, Launay F, Maunder M (2015). The importance and benefits of species. Current Biology.

[CR28] Gusset M, Dick G (2010). “Building a Future for Wildlife”? Evaluating the contribution of the world zoo and aquarium community to in situ conservation. International Zoo Yearbook.

[CR29] Gusset M, Dick G (2011). The global reach of zoos and aquariums in visitor numbers and conservation expenditures. Zoo Biology.

[CR30] Gusset M, Fa JE, Sutherland WJ (2014). A horizon scan for species conservation by Zoos and Aquariums. Zoo Biology.

[CR31] Hoffmann M, Hilton-Taylor C, Angulo A, Böhm M, Brooks TM, Butchart SH, Carpenter KE, Chanson J (2010). The impact of conservation on the status of the world’s vertebrates. Science.

[CR32] Holmes G (2014). What is a land grab? Exploring green grabs, conservation, and private protected areas in southern Chile. The Journal of Peasant Studies.

[CR33] Holmes G (2015). Markets, nature, neoliberalism, and conservation through private protected areas in southern Chile. Environment and Planning A.

[CR34] Howes B, Pither R, Prior K (2009). Conservation implications should guide the application of conservation genetics research. Endangered Species Research.

[CR35] Hsu, A., J. Emerson, M. Levy, A. de Sherbinin, L. Johnson, O. Malik, and M. Jaiteh. 2014. *Environmental Performance Index*. Yale Center for Environmental Law and Policy.

[CR36] Isaac NJB, Turvey ST, Collen B, Waterman C, Baillie JEM (2007). Mammals on the EDGE: conservation priorities based on threat and phylogeny. PLoS ONE.

[CR37] IUCN. 2016. The IUCN Red List of threatened species. Version 2016-1. 10.3897/BDJ.4.e10356 PMC5018116 27660524

[CR38] Jenkins CN, Joppa L (2009). Expansion of the global terrestrial protected area system. Biological Conservation.

[CR39] Juffe-Bignoli D, Brooks TM, Butchart SHM, Jenkins RB, Boe K, Hoffmann M, Angulo A, Bachman S (2016). Assessing the cost of global biodiversity and conservation knowledge. PLoS ONE.

[CR40] Lamoreux, J. F., M. W. McKnight, and R. Cabrera Hernandez. 2015. Amphibian Alliance for Zero Extinction Sites in Chiapas and Oaxaca. International Union for Conservation of Nature xxiv: 320

[CR41] Larsen FW, Turner WR, Brooks TM (2012). Conserving critical sites for biodiversity provides disproportionate benefits to people. PLoS ONE.

[CR42] Leménager T, King D, Elliott J, Gibbons H, King A (2014). Greater than the sum of their parts: Exploring the environmental complementarity of state, private and community protected areas. Global Ecology and Conservation.

[CR43] Martin TG, Nally S, Burbidge AA, Arnall S, Garnett ST, Hayward MW, Lumsden LF, Menkhorst P (2012). Acting fast helps avoid extinction: Acting fast avoids extinctions. Conservation Letters.

[CR44] Mascia MB, Pailler S, Krithivasan R, Roshchanka V, Burns D, Mlotha MJ, Murray DR, Peng N (2014). Protected area downgrading, downsizing, and degazettement (PADDD) in Africa, Asia, and Latin America and the Caribbean, 1900–2010. Biological Conservation.

[CR45] McCarthy DP, Donald PF, Scharlemann JPW, Buchanan GM, Balmford A, Green JMH, Bennun LA, Burgess ND (2012). Financial costs of meeting global biodiversity conservation targets: Current spending and unmet needs. Science.

[CR46] Moss A, Jensen E, Gusset M (2015). Evaluating the contribution of zoos and aquariums to Aichi Biodiversity Target 1: Educational Impacts of Zoo Visits. Conservation Biology.

[CR47] Myers N, Mittermeier RA, Mittermeier CG, da Fonseca GAB, Kent J (2000). Biodiversity hotspots for conservation priorities. Nature.

[CR48] Newbold T, Hudson LN, Arnell AP, Contu S, De Palma A, Ferrier S, Hill SLL, Hoskins AJ (2016). Has land use pushed terrestrial biodiversity beyond the planetary boundary? A global assessment. Science.

[CR49] OECD. 2016. Gross domestic product (GDP) (indicator). doi: 10.1787/dc2f7aec-en.

[CR50] Pritchard DJ, Fa JE, Oldfield S, Harrop SR (2012). Bring the captive closer to the wild: Redefining the role of ex situ conservation. Oryx.

[CR51] Redford KH, Coppolillo P, Sanderson EW, Da Fonseca GAB, Dinerstein E, Groves C, Mace G, Maginnis S (2003). Mapping the conservation landscape. Conservation Biology.

[CR52] Ricketts TH, Dinerstein E, Boucher T, Brooks TM, Butchart SHM, Hoffmann M, Lamoreux JF, Morrison J (2005). Pinpointing and preventing imminent extinctions. Proceedings of the National Academy of Sciences of the United States of America.

[CR53] Rockström, J., W. L. Steffen, K. Noone, ÅAsa Persson, F. S. Chapin III, E. Lambin, T. M. Lenton, M. Scheffer, et al. 2009. Planetary boundaries: exploring the safe operating space for humanity.

[CR54] Rodrigues ASL, Andelman SJ, Bakarr MI, Boitani L, Brooks TM, Cowling RM, Fishpool LDC, da Fonseca GAB (2004). Effectiveness of the global protected area network in representing species diversity. Nature.

[CR55] Rondinini C, Di Marco M, Visconti P, Butchart SHM, Boitani L (2014). Update or outdate: Long-term viability of the IUCN Red List. Conservation Letters.

[CR58] SCBD. 2010. *Decision adopted by the Conference of the Parties to the Convention on Biological Diversity at its tenth meeting. X/2. The Strategic Plan for Biodiversity 2011*–*2020 and the Aichi Biodiversity Targets*. https://www.cbd.int/doc/decisions/cop-10/cop-10-dec-02-en.pdf.

[CR59] SCBD. 2014. *Progress towards the Aichi biodiversity targets: an assessment of biodiversity trends, policy scenarios and key actions: Global biodiversity outlook 4 (GBO*-*4) technical report*. Montreal, Quebec, Canada: Secretariat of the Convention on Biological Diversity (CBD).

[CR56] SCBD. 2016. Convention on Biological Diversity. List of Parties. *Convention on Biological Diversity. *https://www.cbd.int/information/parties.shtml

[CR60] Seto KC, Güneralp B, Hutyra LR (2012). Global forecasts of urban expansion to 2030 and direct impacts on biodiversity and carbon pools. Proceedings of the National Academy of Sciences.

[CR61] Steffen W, Crutzen PJ, McNeill JR (2007). The Anthropocene: Are humans now overwhelming the great forces of nature. Ambio.

[CR62] Stern NH, Treasury HM (2006). Stern review: The economics of climate change.

[CR63] Tapley B, Bradfield KS, Michaels C, Bungard M (2015). Amphibians and conservation breeding programmes: Do all threatened amphibians belong on the ark?. Biodiversity and Conservation.

[CR64] Thornton, D. H., and R. J. Fletcher. 2013. Body size and spatial scales in avian response to landscapes: a meta-analysis. *Ecography*. doi:10.1111/j.1600-0587.2013.00540.x.

[CR65] UNEP-WCMC. 2016. Protected Planet 2014-2015- World Database on Protected Areas dataset. *Protected Planet*.

[CR66] Waldron A, Mooers AO, Miller DC, Nibbelink N, Redding D, Kuhn TS, Roberts JT, Gittleman JL (2013). Targeting global conservation funding to limit immediate biodiversity declines. Proceedings of the National Academy of Sciences.

[CR67] Watson JEM, Dudley N, Segan DB, Hockings M (2014). The performance and potential of protected areas. Nature.

[CR68] Watson JEM, Darling ES, Venter O, Maron M, Walston J, Possingham HP, Dudley N, Hockings M (2015). Bolder science needed now for protected areas. Conservation Biology.

[CR69] Watson JEM, Shanahan DF, Di Marco M, Allan J, Laurance WF, Sanderson EW, Mackey B, Venter O (2016). Catastrophic declines in wilderness areas undermine global environment targets. Current Biology online.

[CR70] Wilson KA, Evans MC, Di Marco M, Green DC, Boitani L, Possingham HP, Chiozza F, Rondinini C (2011). Prioritizing conservation investments for mammal species globally. Philosophical Transactions of the Royal Society B: Biological Sciences.

[CR71] Wilson KA, Auerbach NA, Sam K, Magini AG, Moss ASL, Langhans SD, Budiharta S, Terzano D (2016). Conservation research is not happening where it is most needed. PLoS Biology.

[CR72] Young RP, Hudson MA, Terry AMR, Jones CG, Lewis RE, Tatayah V, Zuël N, Butchart SHM (2014). Accounting for conservation: Using the IUCN Red List Index to evaluate the impact of a conservation organization. Biological Conservation.

[CR73] Zoological Society of London, ZSL. 2016. EDGE of Existence programme

